# Evaluation of emphysema on thoracic low-dose CTs through attention-based multiple instance deep learning

**DOI:** 10.1038/s41598-023-27549-9

**Published:** 2023-01-21

**Authors:** Jordan Fuhrman, Rowena Yip, Yeqing Zhu, Artit C. Jirapatnakul, Feng Li, Claudia I. Henschke, David F. Yankelevitz, Maryellen L. Giger

**Affiliations:** 1 Committee on Medical Physics, Department of Radiology, The University of Chicago, 5841 S Maryland Avenue, MC2026, Chicago, 60637 USA; 2 Department of Radiology, Icahn School of Medicine at Mount Sinai, New York, 10029 USA

**Keywords:** Computed tomography, Translational research

## Abstract

In addition to lung cancer, other thoracic abnormalities, such as emphysema, can be visualized within low-dose CT scans that were initially obtained in cancer screening programs, and thus, opportunistic evaluation of these diseases may be highly valuable. However, manual assessment for each scan is tedious and often subjective, thus we have developed an automatic, rapid computer-aided diagnosis system for emphysema using attention-based multiple instance deep learning and 865 LDCTs. In the task of determining if a CT scan presented with emphysema or not, our novel Transfer AMIL approach yielded an area under the ROC curve of 0.94 ± 0.04, which was a statistically significant improvement compared to other methods evaluated in our study following the Delong Test with correction for multiple comparisons. Further, from our novel attention weight curves, we found that the upper lung demonstrated a stronger influence in all scan classes, indicating that the model prioritized upper lobe information. Overall, our novel Transfer AMIL method yielded high performance and provided interpretable information by identifying slices that were most influential to the classification decision, thus demonstrating strong potential for clinical implementation.

## Introduction

In 2021, the US Preventative Services Task Force (USPSTF) expanded eligibility requirements for lung cancer screening due to programs such as the International-Early Lung Cancer Action Program (I-ELCAP) and the National Lung Screening Trial (NLST)^1–3^. These initiatives found improved patient outcomes through lung cancer screening via low-dose CT (LDCT) acquisition, which provides a clinically useful image at reduced radiation dose to the patient than a standard dose CT scan. In addition to lung cancer, other thoracic abnormalities can be visualized within the CT scan range and opportunistic evaluation of these diseases may be highly valuable^4,5^. Emphysema can be identified on CT scans and shares risk factors with lung cancer, and current literature suggests that presence of emphysema may increase risk of lung cancer development^6–11^. Thus, emphysema evaluation on LDCT is appropriate and desirable. However, a manual assessment for each scan is tedious and often subjective, thus an automatic, rapid computer-aided diagnosis system should be investigated^12,13^.

Multiple instance learning (MIL) is a deep learning scheme commonly used in digital pathology that utilizes weak annotations to train models by evaluation of instances (e.g., CT slices) to form a collective classification decision of a bag (e.g., CT scan)^14^. Wang discussed key MIL schemes, mi-Net and MI-Net, which classify scans based on individual instance classifications and pooled instance representations, respectively^15^. Ilse improved MIL schemes through attention-based multiple instance learning, which utilizes attention mechanisms to identify and more heavily weight key instances of whole slide images for cancer detection^16^.

Deep learning, including MIL schemes, have been utilized to automate emphysema evaluation in standard diagnostic and lung screening CT scans. Humphries utilized a convolutional neural network and long short-term memory architecture to classify visual emphysema pattern on CT and Oh used the same model to compare visual emphysema progression with functional impairment and mortality^17,18^. Negahdar automatically segmented lung volumes on chest CT and classified patches of lung tissue based on visual emphysema pattern to quantify severity^19^. Chepylgina and Orting utilized human-engineered features based on histogram features acquired from filtered lung ROIs in a multiple instance learning scheme to characterize COPD and emphysema, respectively, in low-dose CT scans^20,21^. Tennakoon expanded their work to incorporate deep MIL on 3D LDCT patches to classify emphysema presence^22^.

In our work, we utilize deep MIL with transfer learning and attention-based pooling (Transfer AMIL) to evaluate emphysema in LDCT scans and compare performances in classification of disease.

## Methods

We utilized MIL to characterize emphysema at LDCT presentation through the use of convolutional neural networks (CNN) with transfer learning. In this study, the standard MIL terms “instance” and “bag” are used synonymously with CT slice and scan, respectively.

### LDCT imaging data

The data in this retrospective, HIPAA-compliant study consisted of 865 LDCT scans obtained as part of the International-Early Lung Cancer Action Program. IRB approval and informed consent was waived by the University of Chicago Biological Sciences Division/University of Chicago Medical Center IRB due to deidentification of images prior to obtention and confirm that all experiments were performed in accordance with relevant guidelines and regulations. The image selection criteria were as follows: using the database of all participants enrolled in the Early Lung Cancer Action Program at Weill Cornell Medical College and the Icahn School of Medicine at Mount Sinai between 1992 and July 2017, the most recent LDCT scan of 865 participants with LDCT acquisition were selected that included all participants with moderate emphysema and severe emphysema, and randomly selected the remaining participants among 3696 available participants with no emphysema. Emphysema was evaluated by visual scoring according to previously published criteria which, in brief, categorizes CT scans into mild/moderate categories if less than one-half of the lung volume presents with regions of decreased attenuation and severe if more than one-half has decreased attenuation^23^. The scans were non-gated, non-contrast enhanced, and acquired following the parameters and patient information given in Table 1. A radiologist further characterized the emphysema by dominant phenotype (centrilobular, panlobular, paraseptal) and other pulmonary CT findings non-specific to emphysema utilizing the Fleischner criteria^24^. Imaging data is currently not available publicly.

**Table 1:** Database information.

Number of cases	865 (5 excluded from 865)
Dates of acquisition	1997–1999 (8)
	2000–2004 (161)
	2005–2009 (192)
	2010–2014 (128)
	2015–2017 (326)
	NA (50)
Sex at birth	Male (384) female (431) NA (50)
Smoking status	Current (257) former (469) never (89) NA (50)
Age	Mean (66.8) SD (11.4) range (33–93)
Pack-years of smoking	Mean (36.2) SD (30.8) range (0–199)
Scanner manufacturer	GE medical systems siemens
Exposure time	Range (250–2100)
kVp	(100, 120, 140)
Slice thickness	Range (0.5–10 mm)
Emphysema severity	None (508) mild/moderate (240) severe (117)
Dominant emphysema phenotype	Centrilobular (284) paraseptal (33) panlobular (40)

### Multiple instance learning (MIL)

Typically, MIL is posed as a binary classification problem in which the data are composed into bags ${X}_{i}=\{{x}_{i,1},{x}_{i,2},\dots ,{x}_{i,N}\}$ each of which is composed of *N* instances ${x}_{i,j}$^15,16^. The corresponding instance truths ${y}_{i,j}\in \{\mathrm{0,1}\}$ are unknown, but the bag truth is determined from the instance truths by the binary decision rule

$${Y}_{i}=\left\{\begin{array}{ll}0 & iff \sum_{j}^{N}{y}_{i,j}=0 \\ 1 & otherwise\end{array}\right.$$

MIL can be broken down into three key steps as: (1) Extraction of instance representations, (2) transformation from instance representations to bag representation through MIL pooling, and (3) classification of bag representation for clinically relevant decision^25^. In all, the process is described by

$$\widehat{{Y}_{i}}=g({\varvec{P}}f\left({{\varvec{X}}}_{i}\right))$$

where $\widehat{{Y}_{i}}$ is the predicted bag label, ${{\varvec{X}}}_{i}$ is the set of input CT slices (images) that are transformed to instance representations via $f$, pooled via matrix ${\varvec{P}}$, and transformed to a bag prediction via $g$^25^.

### Transfer learned instance feature extraction (transfer MIL)

In our study, instant representations $f\left({{\varvec{X}}}_{i}\right)$ of CT slices are acquired through transfer learning from a pre-trained VGG19 architecture^26^. Transfer learning utilizes large models with deep, hierarchical features after pre-training for a similar task, in this case image classification but on the ImageNet database set of natural objects^27–29^. In situations where little training data are available, transfer learning allows for the extraction of more complex, rich data representations than can be achieved by training a model from scratch. In this study, we utilized a VGG-19 architecture pre-trained for natural image classification on ImageNet to extract quantitative features similar to the scheme proposed by Antropova^30^.

The instance representations were then input to two fully connected layers with ReLU activation with a dropout rate of 0.5.

### Attention-based MIL pooling (AMIL)

Attention mechanisms have been widely utilized in deep learning to both improve performance and provide interpretability of model predictions^31^. In our study, the pooling matrix ***P*** was constructed through the MIL attention mechanism in which a bag representation was acquired through a weighted average of instance representations:

$${\varvec{z}}=\sum_{n=1}^{N}{a}_{{\varvec{n}}}{{\varvec{x}}}_{{\varvec{n}}} \quad \quad \quad {a}_{n}= \frac{exp \left({{\varvec{w}}}^{T}\mathit{tan}h\left({\varvec{V}}{{\varvec{x}}}_{n}^{T}\right)\right)}{{\sum }_{j=1}^{N}exp \left({{\varvec{w}}}^{T}\mathit{tan}h\left({\varvec{V}}{{\varvec{x}}}_{j}^{T}\right)\right)}$$

for learned parameters ${\varvec{w}}\in {\mathbb{R}}^{128}$ and ${\varvec{V}}\in {\mathbb{R}}^{128 \mathrm{x} 512}$ with *N* input instances ${{\varvec{x}}}_{n}^{T}$ with dimension 512 and hidden dimension 128. The attention weights also provided interpretable output inherent to the decision task in the form of influential instances (i.e., slices), which were evaluated separately for model validation and interpretability.

**Figure 1:**
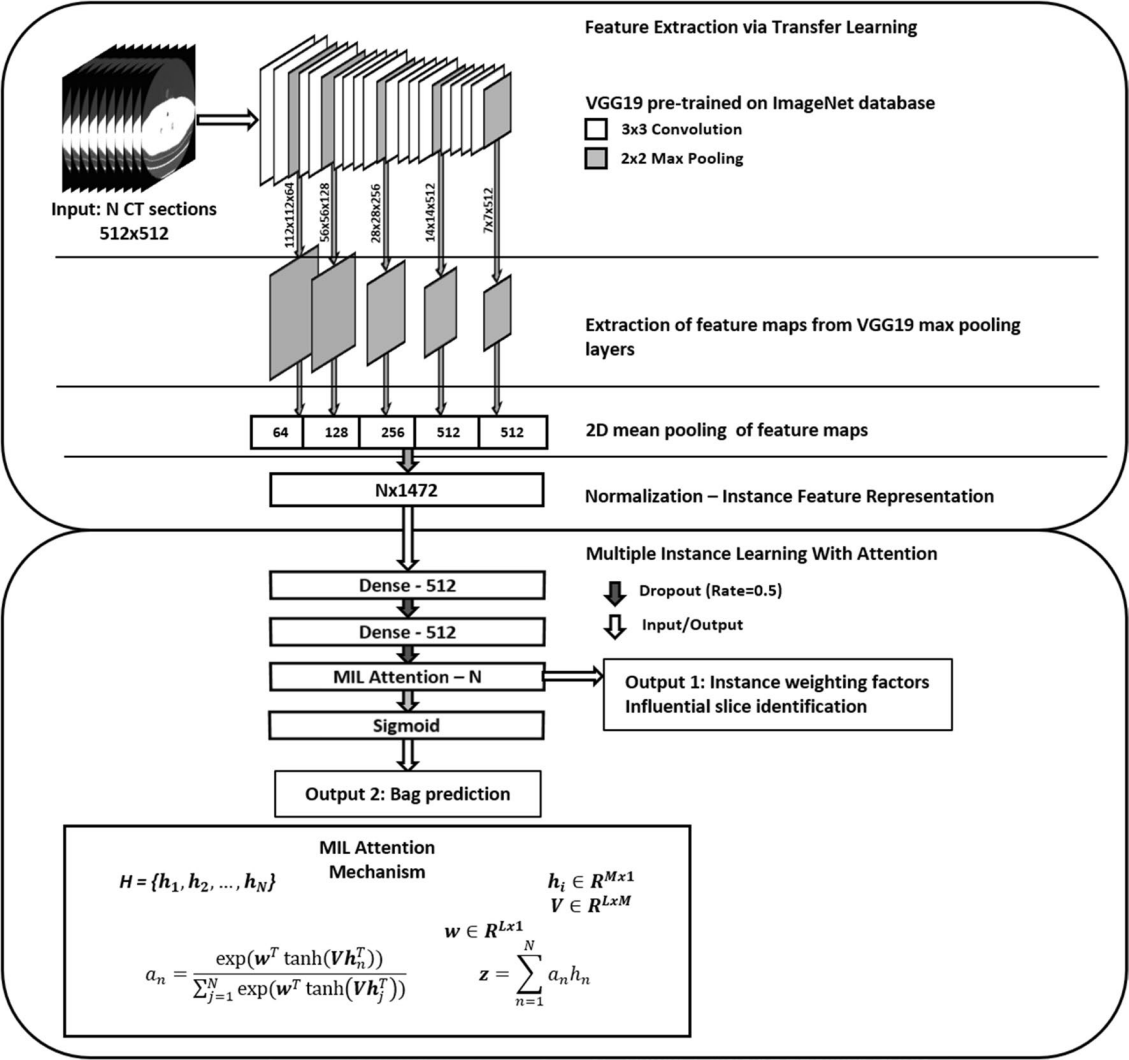
Model workflow of the Transfer AMIL approach. This includes feature extraction of CT images through an ImageNet pre-trained model based on methods developed by Antropova et al. followed by attention-based MIL pooling based on methods developed by Ilse et al. Two outputs are generated for each LDCT scan input, the attention weights which identify influential slices for the classification task and the scan prediction for the presence of emphysema.

The attention weights for different scan classes (dominant emphysema phenotypes of centrilobular, panlobular, and paraseptal) were evaluated by scaling attention weights for a given scan to the range [0, 1] and plotting as a function of the axial depth to determine regions of high and low influence. Influence was quantified by three metrics: (1) depth maximum attention of fit curve, (2) weighted average of slice depths weighted by attention, and (3) range of fit curve attention values. The full workflow of Transfer AMIL is provided in Fig. 1.

### Implementation details

All models were trained in Keras (2.2.4) with Tensorflow backend (2.2.0) in Python (3.7) and optimized by binary cross entropy loss calculated for bag predictions. Adam optimization was utilized with parameters *β*_*1*_ = 0.9 and *β*_*2*_ = 0.99 and initial learning rate of 0.0001. Early stopping was initiated if the validation loss did not improve after 7 epochs. All learned parameters were initialized by sampling a normal distribution.

### Training, testing, and statistical evaluation

Models were trained with 70%, 10%, and 20% of the available cases serving for training, validation, and testing, respectively, repeated 5 times with different randomly generated splits. The mean and variance of the area under the ROC curve (AUC) were obtained across the five models. AUCs were compared through the Delong test on each of the five training passes with the median *p*-value serving as the metric for significance^32^.

### Compared methods

We compared Transfer AMIL to other approaches which required only scan annotations. A 3D CNN classifier was trained by interpolating to a fixed input size of 128 slices and scan presence of emphysema serving as binary class. Additionally, a standard 2D classifier was trained by assigning the scan ground truth class to all slices within the scan regardless of emphysema presence within that slice; this caused noisy labels during training, particularly with many false positive slices for severe emphysema cases.

## Results

### Binary classification performance

In the task of determining if a CT scan presented with emphysema or not, the Transfer AMIL approach yielded an area under the ROC curve of 0.94 ± 0.04, which was a statistically significant improvement compared to other methods evaluated in our study following the Delong Test with correction for multiple comparisons (Table 2). Transfer AMIL performed better than or similar to other published work, including shallow, human-engineered MIL methods, as well as other deep MIL approaches, although it is important to note that others’ evaluations were on different datasets.

**Table 2:** Performance assessment.

Algorithm	AUC from ROC analysis	Human-engineered features	Deep CNN features	Interpretable	Transfer learning
Transfer AMIL	0.94 ± 0.04		X	X	X
Noisy 2D Classifier	0.85 ± 0.06		X		
Fully 3D Classifier	0.58 ± 0.16		X		
AMIL	0.69 ± 0.05		X	X	
Mean pooling	0.90 ± 0.02		X		X
Max pooling	0.88 ± 0.02		X		X
Cheplygina^20^	0.78 ± 0.04	X		X	
Orting^21^	0.88 ± –	X			
Tennakoon^22^	0.95 ± –		X		

### Attention weights across emphysema classes

**Figure 2:**
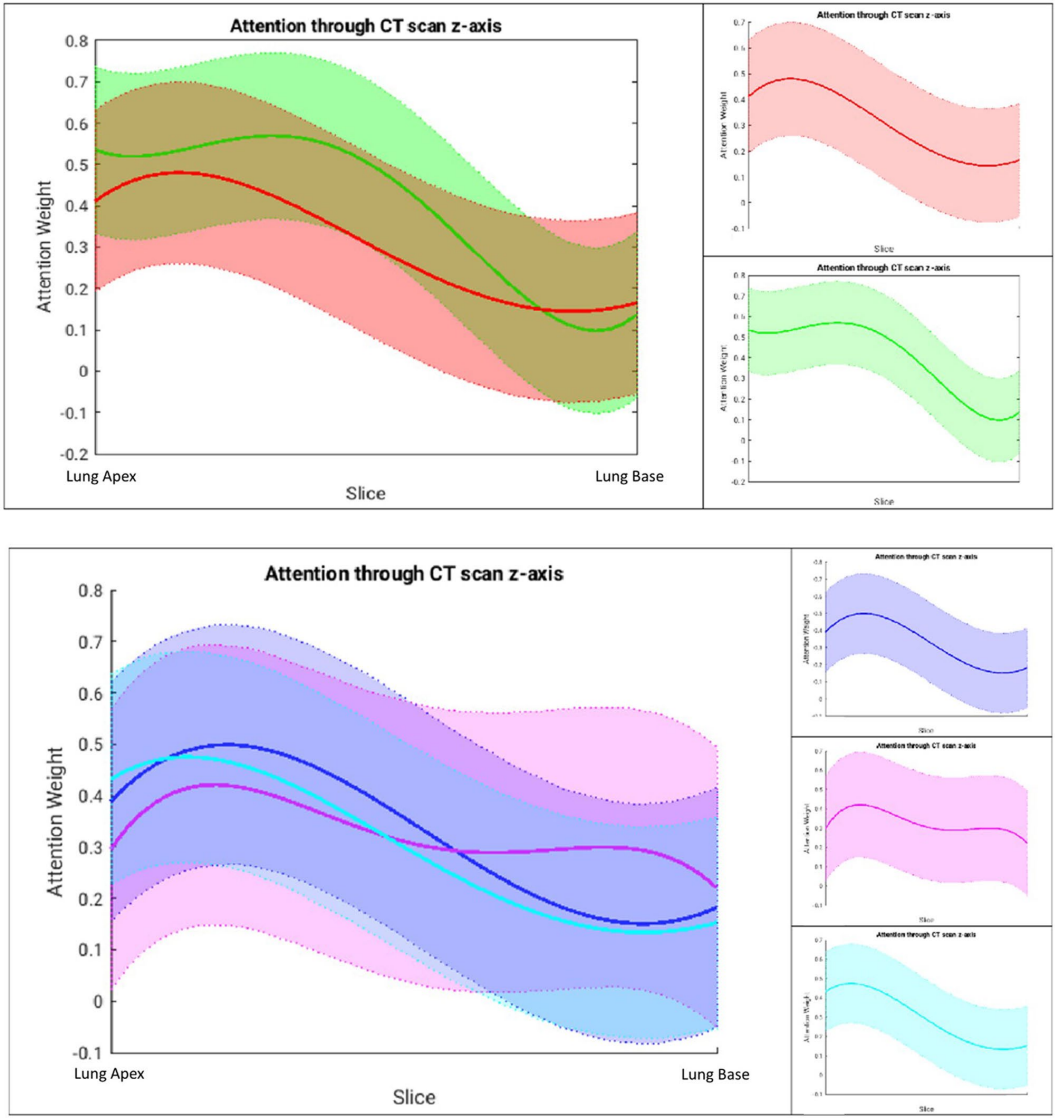
Attention weight curves illustrating the fit of attention weights from CT slices as a function of height in the lungs for (top) positive (red) and negative (green) LDCT scans and (bottom) for different dominant phenotypes of emphysema: centrilobular (blue), panlobular (pink), and paraseptal (turquoise). Since patients’ CT scans have variable number of slices covering the lung region, in these plots, the range has been normalized to fit between Lung Top and Lung Bottom.

Attention weight curves were calculated to demonstrate the influence of disease type localized throughout the lung. The attention weights demonstrated a stronger influence for slices in the upper lung in all scan classes, indicating that the model prioritized upper lobe information (Table 3, Fig. 2). This agrees with published literature trends that note an upper lobe predominance for emphysema, particularly centrilobular, the most common phenotype in this dataset^33–36^. Recall, influence is quantified by three metrics: (1) depth maximum attention of fit curve, (2) weighted average of slice depths weighted by attention, and (3) range of fit curve attention values.

**Table 3:** Quantitative attention weights from Fig. 2.

Scans evaluated	Maximum attention lung depth (%)	Weighted average of lung depth (%)	Range of attention values (%)
Positive scans	15.6	38.5	33.6
Negative scans	32.9	38.7	47.2
Positive: centrilobular	19.3	39.1	34.8
Positive: panlobular	17.2	46.2	20.0
Positive: paraseptal	12.8	37.6	34.1

Recall, influence is quantified by three metrics: (1) depth maximum attention of fit curve, (2) weighted average of slice depths weighted by attention, and (3) range of fit curve attention values.

By phenotype, the centrilobular and paraseptal attention average depths (39.1%, 37.6%) aligned with expected upper lobe predominance compared to panlobular (46.2%). Further, the panlobular scans tended to more heavily influence slices throughout the lung range as demonstrated by the reduced range of attention values (20.0%) compared to the other phenotypes (34.8%, 34.1%). Note that any given scan did not necessarily present with only one phenotype; for example, the scans labeled panlobular-dominant may also present with other phenotypes. This and the model’s learned predisposition to more highly weight the upper lobe slices (as conveyed by the quantification of negative scan attention) may account for the relative importance of the upper lobes even in the panlobular-dominant scans.

### CT image features emphasized by attention weights

**Figure 3:**
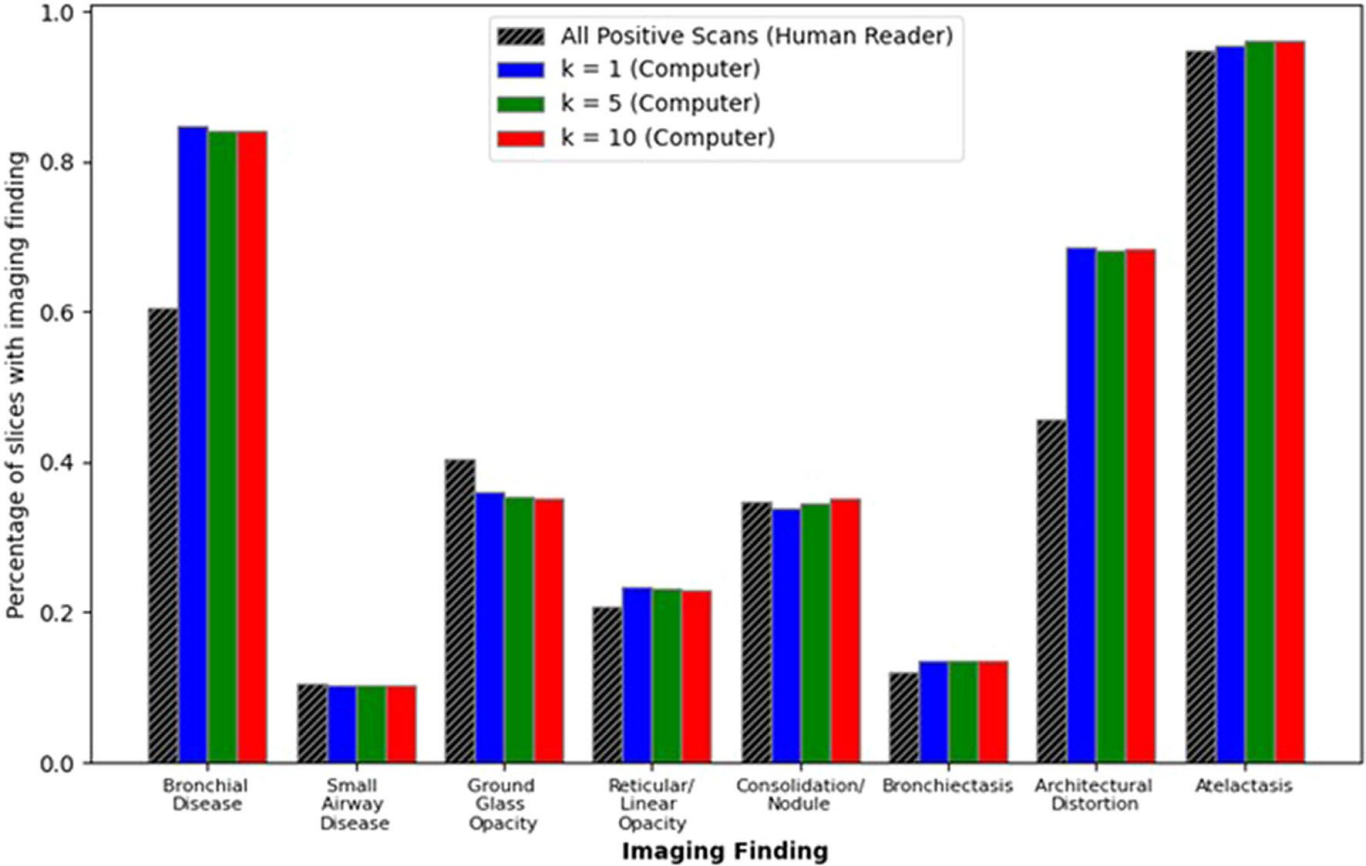
Evaluation of common thoracic imaging features. The prevalence of each feature within the entire CT scan as identified by a radiologist and when selected by the top-*k* attention weighted slices. Note key differences between whole slice prevalence and selected prevalence: bronchial disease and architectural distortions were more heavily weighted while ground glass opacities are diminished. Further, the consistent representation across the top-*k* slices for different *k* demonstrates the model’s tendency to more heavily weight slices with similar extracted representations.

The top-*k* influential slices according to attention weights were evaluated to determine which CT imaging features drew the most attention and to identify potential sources of misclassification. The prevalence of image features that were present in the top-*k* selected attention weighted slices are shown in Fig. 3. Different features were likely to have different prevalence within each scan (e.g., nodules were local abnormalities while architectural distortions were generally more widespread structural changes), thus the prevalence of each imaging feature as identified by a radiologist is presented for comparison. Bronchial disease and architectural distortions demonstrated the largest change in importance for the top-*k* attended slices compared to the human reader with changes of 23.7% ± 0.03% and 22.7% ± 0.01%, respectively. The prevalence of each feature did not significantly change when including more slices in the attention analysis; however, bronchial disease and architectural distortion features were attended to much more frequently than their frequency in entire scans while the opposite occurred for ground glass opacities. This may suggest that the model was balanced between identifying features indicative of emphysema presence, such as regions of hypoattenuation and structural changes, while maintaining a general representation of the entire CT scan.

## Discussion

In this study, we present a novel CT slice-based Transfer AMIL approach for evaluating emphysema on LDCT scans acquired for lung cancer screening. The model provides strong classification performance compared to models with similar label constraints, including models evaluated for this study and those published in the literature. The attention module also provides interpretable information for verifying model performance by identifying slices that were most influential to the classification decision. Indeed, the attention weight trends for different subsets of the LDCT scans agreed with expectations in terms of the most likely regions to find emphysema, including when different classes of emphysema were dominant. A further investigation into the attention weights also revealed which CT image features were most useful for the model prediction and may provide insight into what potential cases will be problematic for automatic evaluation, particularly considering the lung cancer screening population.

Importantly, the developed model requires a relatively small amount of computing power compared to other modern deep learning computer vision tasks. The AUC performance achieved by the Transfer AMIL was either comparable or outperformed other models in this study, including standard 2D classification models with noisy labels and 3D image classifiers. Note that this performance may only hold true for the data available for this study; a large amount of data would likely improve the non-transfer learning models more than transferred models because during training the number of training images approaches the number of trainable parameters.

The pre-trained VGG19 feature extraction model parameters (20 M) were fixed from the pre-training task with no additional training; additional training would further underdetermine the model considering the limited dataset of 860 utilized scans (of 865 total with 5 excluded). With the feature extractor fixed, the additional fully connected layers and attention module require only 1.15 M trainable parameters; still an underdetermined system, but at a greatly reduced risk of overfitting. While larger standard architectures such as ResNet50 and DenseNet121 could be utilized for feature extraction, this study demonstrates that even the use of smaller, less complex models can achieve competitive performance. Note, these architectures were evaluated but no performance gain was observed thus the least computationally expensive model was utilized. Further, the reduced model capacity and use of transfer learning with a common architecture encourage wider implementation of this technique because the compute power needed to run the model is generally attainable by today’s standards and feature extractor does not require local training.

While the attention module interpretable output validates model performance compared to literature, it also encourages clinical implementation as the attention weights can be added as an optional part of the lung screening workflow for a radiologist to further investigate the classification decision, specifically by review of the slices influential to the classification decision. This review process can lead to radiologist trust and understanding of clinical implementation of the algorithm and has the potential to improve clinical workflow in terms of both reading time and performance, although this would require a prospective reader study to confirm.

The theme of improved performance also aligns with the attention module’s ability to identify which cases may be problematic for classification. For example, the model tended to more heavily weight slices with bronchial disease and architectural distortions, which are nonspecific to emphysema patients, and which often appear similar to typical presentation of emphysema (e.g., regions of hypoattenuation and structural changes). This also suggests that patients with these presentations caused by non-emphysematous conditions may be difficult for the model to classify.

Future work should prospectively utilize this model in a reader study to evaluate its impact on radiologist performance and radiological workflow as well as include images acquired from multiple institutions to assess model generalizability. This is especially important as the data in this study were limited (single institution, limited *N*). Further, this study only evaluated binary classification decisions and does not consider relationships between slices when calculating attention weights; multi-class variants of MIL as well as more complex attention-based pooling functions. Despite these limitations, the Transfer AMIL method achieved strong performance as determined from ROC analysis and the attention weight investigations performed in this study demonstrated strong potential for clinical implementation.

## Data Availability

The data generated and analyzed during this study may be made available by contacting the corresponding author with reasonable request.
